# Parental influences on adolescent physical activity: a longitudinal study

**DOI:** 10.1186/1479-5868-4-3

**Published:** 2007-02-02

**Authors:** India J Ornelas, Krista M Perreira, Guadalupe X Ayala

**Affiliations:** 1 Department of Health Behavior and Health Education and Carolina Population Center, University of North Carolina at Chapel Hill, CB#7440, Chapel Hill, NC 27599, USA; 2 Department of Public Policy and Carolina Population Center, University of North Carolina at Chapel Hill, CB#3435, Chapel Hill, NC 27599, USA; 3 Center for Behavioral and Community Health Studies, Graduate School of Public Health, San Diego State University, San Diego, CA 92123, USA

## Abstract

**Background:**

Physical inactivity is increasing among adolescents in the U.S., especially among girls. Despite growing evidence that parents are an important influence on adolescent health, few longitudinal studies have explored the causal relationship between parental influence and physical activity. This study examines how the relationships between parental influences and adolescent physical activity differ by gender and tests whether these relationships are mediated by adolescents' self-esteem and depression.

**Methods:**

Data are from the National Longitudinal Study of Adolescent Health. The sample includes 13,246 youth, grades 7 to 12, interviewed in 1995 and again 1 year later. Logit models were used to evaluate parental influences on achieving five or more bouts of moderate to vigorous physical activity per week [MVPA] and whether the relationship between parental influence and MVPA was mediated by adolescents' level of self-esteem and depression.

**Results:**

Family cohesion, parent-child communication and parental engagement positively predicted MVPA for both genders one year later (odds ratios and 95% confidence intervals for females, 1.09 [1.05–1.12], 1.13 [1.07–1.19], 1.25 [1.17–1.33] and males, 1.08 [1.04–1.11], 1.14 [1.07–1.23], 1.23 [1.14–1.33], respectively); however, parental monitoring did not (odds ratio and confidence intervals for females and males, 1.02 [.97–1.07]). For both females and males, self-esteem mediated the relationship between parental influence and physical activity. Depressive symptoms were only a mediator among males. Females reported higher levels of parent-child communication and lower family cohesion compared with males. There were no gender differences in levels of parental monitoring and engagement. Females had significantly lower levels of self-esteem and higher levels of depressive symptoms than males.

**Conclusion:**

Strategies to promote physical activity among adolescents should focus on increasing levels of family cohesion, parental engagement, parent-child communication and adolescent self-esteem.

## Background

Low levels of physical activity during adolescence contribute to obesity and poor health outcomes in adulthood [1-3]. Physical inactivity is increasing among adolescents in the U.S., especially among girls [4-7]. In 2005, over 30% of adolescents failed to meet national recommendations for moderate to vigorous physical activity [8].

During childhood and adolescence, families critically influence their children's health behaviors including physical activity [6,9-13]. Parents model behaviors for their children, engage in activities with them, monitor their children's behaviors, and provide support and encouragement that can result in behavior change and positive health outcomes [14-16]. Despite the centrality of family influences on child development, few studies have examined the causal relationship between family-related factors and adolescent physical activity [17,18]. An understanding of these relationships is essential to the development of family-focused interventions programs that promote adolescent physical activity, and ultimately prevent overweight and obesity.

Previous research has identified parental social support and modeling as important influences on child and adolescent physical activity [6,19-22]. Broader aspects of parental influences, such as parental engagement, monitoring, and communication remain understudied, despite significant research concerning their influences on adolescent risk behaviors such as tobacco and alcohol use [23,24]. Several mechanisms through which parenting can influence adolescent physical activity have been proposed [25]. One potential mediator of parental influence on adolescent physical activity is adolescent emotional health [26,27]. Parenting behaviors influence low self-esteem and depression, both of which are inversely associated with physical activity [28-33]. Finally, previous research has shown that boys engage in higher levels of physical activity and receive more parental support for physical activity than girls [21,34,35]. However, individuals at risk of low levels of physical activity (e.g. girls) may be more responsive to parental influences. Therefore, gender differences in the relationship between parental influences and physical activity must be critically examined.

Using longitudinal data from a large nationally representative sample, this study examined the relationship between parental influences and adolescent physical activity by gender and tested whether these relationships were mediated by adolescents' self-esteem and depression. It was hypothesized that high levels of family cohesion, parental monitoring, parent-child communication, and engagement in activities would predict higher levels of physical activity one year later and that the influence of these relationships would be mediated by adolescent self-esteem and depressive symptoms. Given inconsistent results in previous research, gender differences in the relationship between family and parental influences and physical activity were hypothesized, but it was unclear whether family and parental influences would be stronger for boys or girls.

## Methods

### Study design

This study used data from the first and second waves of the National Longitudinal Study of Adolescent Health (Add Health), a nationally representative study of adolescents in grades 6 through 12 in the U.S. Add Health used a multi-stage, stratified, school-based, cluster sampling design, which ensured that the sample of 80 high schools and their corresponding middle schools was representative of the U.S. population [36,37]. Further details on the survey and sampling design have been extensively described elsewhere [36-38].

Wave I in-home interviews were conducted from April to December 1995 with a random sample of 20,745 students. The sample included approximately 200 students from each high school, middle school pair [37]. With the exception of students with disabilities or those included in a special genetic subsample, all 7^th ^to 12^th ^graders were re-interviewed one year later [Wave II]. A total of 14,736 adolescents participated in both Wave 1 and Wave II interviews. Response rates for Wave I and II in-home interviews were 79% and 88%, respectively.

Because the study design requires that cases be weighted to control for the sampling design, only cases with an assigned sampling weight were included in our final analytic sample (N = 13,246). Due to their small sample size, 29 female and 38 male Native Americans were excluded from this analysis. We also excluded adolescents who were currently pregnant in either Wave I or II, adolescents with serious disabilities, and those with missing data on key demographic variables. Detailed analyses of non-response bias and missing data showed that biases remaining after adjusting for sampling weights were very small (<1%) [39].

### Measures

#### Physical activity

Measured at Wave II, the primary outcome of interest was total weekly bouts of moderate to vigorous physical activity [MVPA]. This outcome, examined in several previous studies [38,40,41], was derived using a standard seven-day physical activity recall scale similar to those used in other large-scale studies although not capturing time period [42-44]. Questions were worded, "During the past week, how many times did you...," followed by a list of activities, allowing calculation of the number of physical activity bouts per week. MVPA activities included skating, cycling, exercise and active sports, and had an estimated energy cost of five to eight METs (metabolic equivalent values; 1 MET = resting metabolic rate, or 3.5 ml 0_2 _body weight/minute). Overall physical activity frequency was summed to obtain total weekly bouts of MVPA. Then, an indicator variable (1/0) was created based on whether the adolescent met the 1995 national recommendations for physical activity. In 1995, the Centers for Disease Control and Prevention and the American College of Sports Medicine recommended engaging in five or more weekly bouts of at least 30 minutes of MVPA [45]. Newly released recommendations for children and adolescents have increased to 60 minutes of moderate activity most days of the week [46].

#### Family cohesion and parenting

Four characteristics were examined: (1) family cohesion, (2) parental monitoring, (3) parent-child communication, and (4) parental engagement. All were measured at the baseline interview in Wave I and have been used in previous studies [47-51]. Family cohesion was measured by summing responses to adolescent reports for three items (ranging from 1 = low to 5 = high) on how much people in their family understand them, how much they and their family have fun together, and how much their family pays attention to them (alpha = .79). Parental monitoring was measured by a sum of seven household rules and guidelines. These included weekday and weekend curfews, monitoring friendships, limiting the amount and type of TV shows watched, controlling food choice, and setting guidelines on appropriate clothing. Higher scores indicated higher levels of parental monitoring (range 0 = low to 7 = high; alpha = .61). Parent-child communication was calculated as the sum of three types of communication that the adolescent had with his/her primary caregiver in the last four weeks (talking with them about dating, a personal problem and school work; range 0 = low to 3 = high; alpha = .54). Parental engagement was measured as a count of the number of activities that parents participated in with their adolescent over the last four weeks. Six possible activities included attendance at religious or church events, shopping, playing sports, going to movies, plays, museums, concerts or sports events, working on a school project, and eating evening meals with their child at least five days a week. For adolescents with monitoring, communication, and engagement scores for both a mother and father, the scores were averaged across the two parents. The highest correlation between these measures was .49 [see Additional file 1]. Due to the multicollinearity between family influences, we estimated their effects both independently and together.

#### Self-esteem and depression

Measures of self-esteem and depressive symptoms were collected during the adolescent in-home interview at Wave I. Self-esteem was measured using the 6-item personal self-image scale created by Resnick and collegues (alpha = .85) [29,52]. Items were reverse coded and summed with possible scores ranging from 0 – 24, so that lower scores indicate lower self-esteem. Depressive symptoms were measured using a slightly modified version of the Center for Epidemiological Studies' Depression Scale (CES-D) [53,54]. A score for this scale is created by summing responses to how often the adolescents experienced 19 different symptoms (e.g. poor appetite, feeling tired) within the past week (alpha = .87). Response categories range from 0 = never or rarely to 3 = most or all of the time. An indicator variable (1/0) was created based on whether the adolescent had a score of 16 or higher. As with the standard CES-D, a score of 16 or above is positively and strongly correlated with a diagnosis of clinical depression [55].

#### Other covariates

Several variables were included in our analyses as covariates, including the adolescent's age at Wave I, their self-reported racial or ethnic group (i.e., European/Canadian, African-American, Mexican, Cuban, Central and South American, Puerto Rican, Chinese, Filipino, and Other Asian), and their immigrant generation status (i.e. foreign-born [1^st ^generation], foreign-born with at least one U.S. born parent [2^nd ^generation], or U.S. born with U.S. born parents [3^rd+ ^generation]). Family structure was dummy-coded based on 5 categories: two-parent family (including biological or adoptive parents), step-parent family, single-mother household, single-father household, or other (e.g., adolescents in foster families or group homes, and emancipated minors). Number of siblings living at home was also included. Parental education, which served as a proxy for the socioeconomic status of the adolescents' household, was measured as the highest level of education achieved by either parent. Adolescents' body mass index (BMI) at Wave 1 was also included as a control in the initial analyses; however, we removed it due to the number of cases with missing BMI data [see Additional file 2]. This exclusion did not change the results.

### Statistical analyses

Analyses were conducted with the statistical software package STATA, version 8.0 (Statacorp, College Station, TX, 2003) and survey estimation procedures were used to correct for the sample design and use of sampling weights. Descriptive statistics including frequencies, means and standard errors were calculated for all study variables. We tested for differences in proportions of those with 5 or more bouts of MVPA per week by gender, race-ethnicity, immigrant generation status, family structure, and parental education at Wave I.

Because our dependent variable is dichotomous, a series of logit models were estimated to identify the likelihood that adolescents engaged in 5 or more bouts of MVPA per week. We exponentiated the estimated coefficients in each model and reported odds ratios. An odds ratio greater than 1.0 indicates that the outcome is more likely in the presence of the independent variable versus in the absence of the independent variable. An odds ratio less than 1.0 indicates an outcome is less likely in the presence of the independent variable versus in the absence of the independent variable. First, models were estimated for each independent variable individually in order to observe the effects of each parental influence independently. Then models were estimated with all parental influence variables entered together. Tests for the mediation by self-esteem and depression were conducted following the criteria outlined by Barron and Kenny [56]. All analyses were stratified by gender. In addition, all analyses were adjusted for age, race-ethnicity, immigrant generation, family structure, number of siblings, and parents' level of education.

Each of the four parenting variables contained a small number (< 100) of missing values. Following Allison, we conducted analyses with missing cases excluded (analyses not shown) [57]. We then re-estimated each model with missing data replaced by mean substitution, and included an indicator variable for these cases. The results from the two estimations were similar and suggested the few cases with missing data were not particularly influential. In light of this finding and the small number of cases, more sophisticated techniques of dealing with missing data were not warranted.

## Results

Table 1 shows the characteristics of the sample and the proportion of adolescents meeting 1995 national recommendations for physical activity by racial/ethnic group, immigrant generation, family structure and parent's level of education. The sample was 51% female with a mean age of 15.5 at Wave I. A higher percentage of males (72%) met the 1995 national recommendations for physical activity than females (58%). White female adolescents were significantly more likely to achieve five or more bouts of MVPA per week than females in other racial and ethnic groups (F-statistic 19.53, p ≤ .001); African-American females were significantly less likely to achieve five or more bouts than other racial and ethnic groups (14.13, p ≤ .001). No racial and ethnic differences were observed among male adolescents. Among females, first generation immigrants were less likely than second or third+ generation immigrants to achieve five or more bouts of MVPA (F-statistic 8.3, p ≤ .01). A similar trend was observed among male adolescents although the relationship was not statistically significant. There were no differences between two parent, step-parent, or single parent families. However, those in the "other" family structure were less likely to achieve recommended levels of MVPA than those in two parent, step-parent or single parent families (F-statistic for males 3.94, p ≤ .05; females 15.63, p ≤ .001). Adolescents in "other" family structures are mostly living in foster care arrangements. Having a parent with at least a college degree was significantly associated with engaging in five or more bouts of MVPA for both females and males (F-statistic for males and females 19.5, p ≤ .001).

**Table 1:** Sample characteristics and percentage of adolescents achieving recommended levels of physical activity

	Females (N = 6,730)		Males (N = 6,561)	
		
	N	%	N	%
**Race-Ethnicity**				
White	3,654	60.8	3,541	72.7
African-American	1,485	49.4	1,307	74.4
Hispanic/Latino	1,105	52.8	1,141	70.9
Asian-American	486	52.5	527	70.3
**Immigrant Generation**				
Third or More	5,246	58.2	5,027	72.5
Second	965	58.9	720	75.1
First	519	47.2	493	68.7
**Family Structure**				
Two-parent family	3,573	58.9	3,516	73.5
Step-parent family	1,124	60.3	1,177	71.1
Single mother	1,538	55.3	1,290	73.2
Single father	152	59.7	238	70.2
Other	343	42.9	295	66.4
**Parent Education**				
College graduate or more	2,280	61.6	2,389	77.2
Some college	1,354	60.0	1,216	70.9
High school graduate	1,896	55.4	1,834	71.5
Less than high school graduate	870	53.7	735	67.4

Table 2 displays the means and standard errors for the study variables by gender. Average activity levels for the study sample met 1995 national recommendations of five or more bouts per week. In terms of parent factors, there were no gender differences for levels of parental monitoring and parental engagement. However, females reported higher levels of parent-child communication and lower levels of family cohesion compared with males. Females had significantly lower levels of self-esteem and higher levels of depressive symptoms than males.

**Table 2:** Descriptive statistics for study variables

	Females (N = 6,730)		Males (N = 6,516)		Gender Differences
			
	Mean	(s.e.)	Mean	(s.e.)	
**Physical Activity**					
Bouts of physical activity per week	6.03	(0.11)	7.88	(0.11)	***
**Parental Influence**					
Family Cohesion (range 3 – 15)	11.18	(0.06)	11.42	(0.06)	***
Parental Monitoring (range 0 – 7)	2.02	(0.05)	2.00	(0.06)	
Parent – Child Communication (range 0 – 3)	1.90	(0.03)	1.65	(0.03)	***
Parental Engagement (range 0 – 6)	2.15	(0.04)	2.12	(0.04)	
**Adolescent Emotional Health**					
Self-Esteem (range 0 – 24)	20.83	(0.11)	22.32	(0.09)	***
Depression (range 0 – 57)	12.47	(0.17)	10.62	(0.14)	***

*** p ≤ .001

The partially adjusted logit models revealed that family cohesion (OR: 1.09, CI: 1.05 – 1.12), parent-child communication (OR: 1.13, CI: 1.07 – 1.19) and parental engagement (OR 1.25, CI: 1.17 – 1.33) were all independent predictors of achieving five or more bouts of MVPA at Wave II (Table 3). The strength of the relationship was similar for both males and females. Parental monitoring was not associated with physical activity for either gender. Adolescents with higher levels of self-esteem and lower levels of depressive symptoms were more likely to achieve five or more bouts of MVPA per week at Wave II. Lower levels of depressive symptoms were more strongly associated with physical activity for male adolescents.

**Table 3:** Partially Adjusted logits on moderate to vigorous physical activity, by gender^a^

	Females (N = 6,730)			Males (N = 6,516)		
				
	OR	95% CI		OR	95% CI	
**Parental Influence**						
Model 1: Family Cohesion	1.09	(1.05 – 1.12)	***	1.08	(1.04 – 1.11)	***
Model 2: Parental Monitoring	1.02	(0.97 – 1.07)		1.02	(0.72 – 0.79)	
Model 3: Parent-Child Communication	1.13	(1.07 – 1.19)	***	1.14	(1.07 – 1.23)	***
Model 4: Parental Engagement	1.25	(1.17 – 1.33)	***	1.23	(1.14 – 1.33)	***
**Adolescent Emotional Health**						
Model 5: Self-Esteem	1.06	(1.04 – 1.08)	***	1.10	(1.07 – 1.12)	***
Model 6: Depression	0.82	(0.70 – 0.96)	**	0.73	(0.72 – 0.79)	***

*** p ≤ .001, ** p ≤ .01

^a ^All models are adjusted for age, race-ethnicity, immigrant generation, family structure, number of siblings, and parent education. Each parental influence and adolescent emotional health variable was entered into the model separately. In addition, models for females and males were estimated separately. Estimates were adjusted for survey design effects.

Table 4 shows results for six fully adjusted logit models constructed to test for the mediational effects of self-esteem and depressive symptoms on the relationship between parental influences and MVPA. In Models 1–6, all parent variables were entered into the model along with covariates. Although parental monitoring was not significantly associated with MVPA in our initial analyses, we included it in these analyses because it was a potential confounder. Using Baron and Kenny's criteria for mediation, we first estimated the effect of parental influences on physical activity. Higher levels of family cohesion, parent-child communication and parental engagement continued to independently predict five or more bouts of MVPA for both males and females when entered together. Parental engagement was the strongest predictor with an odds ratio of 1.19 (95% CI: 1.11, 1.27) for females and 1.18 for males (95% CI: 1.09, 1.27). Overall, we found few differences in the logit models of physical activity between the male and female samples.

**Table 4:** Fully adjusted logits on moderate to vigorous physical activity, by gender^a^

	**Model 1**			**Model 2**			**Model 3**		
**Panel A: Female Sample**	OR	95% CI		OR	95% CI		OR	95% CI	
						
**Parental Influence**									
Family Cohesion	1.05	(0.00 – 1.09)	**	1.02	(0.99 – 1.06)		1.05	(1.02 – 1.09)	**
Parental Monitoring	1.01	(0.96 – 1.05)		1.01	(0.97 – 1.06)		1.01	(0.96 – 1.05)	
Parent-Child Communication	1.06	(1.00 – 1.12)	*	1.05	(0.99 – 1.11)		1.06	(1.00 – 1.12)	*
Parental Engagement	1.19	(1.11 – 1.27)	***	1.18	(1.10 – 1.26)	***	1.19	(1.11 – 1.27)	***
**Adolescent Emotional Health**									
Self-Esteem	---	---	---	1.04	(1.02 – 1.06)	***	---	---	---
Depression	---	---	---	---	---	---	0.94	(0.80 – 1.10)	
N =	6,730			6,730			6,730		
F(29, 99)	10.48			10.38			10.10		
									
**Panel B: Male Sample**	**Model 4**			**Model 5**			**Model 6**		
						
**Parental Influence**									
Family Cohesion	1.05	(1.02 – 1.09)	**	1.01	(0.97 – 1.04)		1.04	(1.01 – 1.08)	*
Parental Monitoring	1.01	(0.95 – 1.06)		1.02	(0.96 – 1.08)		1.01	(0.95 – 1.07)	
Parent-Child Communication	1.08	(1.01 – 1.16)	*	1.07	(0.99 – 1.14)		1.08	(1.01 – 1.17)	*
Parental Engagement	1.18	(1.09 – 1.27)	***	1.16	(1.07 – 1.25)	***	1.17	(1.09 – 1.27)	***
**Adolescent Emotional Health**									
Self-Esteem	---	---	---	1.09	(1.06 – 1.11)	***	---	---	---
Depression	---	---	---	---	---	---	0.79	(0.64 – 0.95)	*
N =	6,516			6,516			6,516		
F(29, 100)	7.62			9.18			7.59		

*** p ≤ .001, ** p ≤ .01, * p ≤ .05

^a ^All models are adjusted for age, race-ethnicity, immigrant generation, family structure, number of siblings, and parent education. In addition, models for females and males were estimated separately. Estimates were adjusted for survey design effects.

Second, we tested for the effect of parental influences on the hypothesized mediators, self-esteem and depression. As shown in Table 5, family cohesion, parent-child communication and parental engagement were significantly associated with increased adolescent self-esteem at the p ≤ .05 level when entered into self-esteem models. Family cohesion, parental monitoring and parental engagement were also significantly associated with lower levels of depressive symptoms at the p ≤ .05 level for models of depressive symptoms.

**Table 5:** Regression coefficients for family environment variables on self-esteem and depression, by gender

	**Females**			**Males**		
				
	**Coefficient**	**95% CI**		**Coefficient**	**95% CI**	
**Self-Esteem**						
Model 1: Family Cohesion	0.75	(0.70 – 0.81)	***	0.58	(0.53 – 0.64)	***
Model 2: Parental Monitoring	-0.05	(-0.15 – 0.04)		-0.10	(-0.18 – -0.02)	*
Model 3: Parent-child Communication	0.49	(0.37 – 0.61)	***	0.43	(0.32 – 0.55)	***
Model 4: Parental Engagement	0.77	(0.65 – 0.88)	***	0.57	(0.46 – 0.68)	***
						
**Depression**						
Model 5: Family Cohesion	-0.27	(-0.31 – -0.24)	***	-0.21	(-0.25 – -0.17)	***
Model 6: Parental Monitoring	0.06	(0.02 – 0.08)	*	0.09	(0.03 – 0.15)	***
Model 7: Parent-child Communication	0.07	(-0.13 – 0.00)	*	0.03	(-0.04 – 0.11)	
Model 8: Parental Engagement	-0.08	(-0.31 – -0.17)	*	-0.14	(0.22 – 0.06)	***

*** p ≤ .001, ** p ≤ .01, * p ≤ .05

Finally, we estimated separate logit models on physical activity controlling for self-esteem and depression (Models 2, 3, 5, and 6). In Models 2 and 5, parental engagement and self-esteem significantly predicted five or more bouts of MVPA at Wave II and the effect of family cohesion and parent-child communication became non-significant for both male and female panels. When depression was added to the original model (Models 1 and 4), family cohesion, parent-child communication and parental engagement remained significant predictors of MVPA at the levels present in the original models (Models 3 and 6). According to the criteria, the effect of family cohesion and parent-child communication on physical activity was mediated by self-esteem for both males and females and the effect of family cohesion was mediated by depression for males.

## Discussion

Parents play an important role in the development of healthy lifestyle behaviors in their children. The results of our study show that parents have a significant influence on adolescent physical activity. Family cohesion, parent-child communication and parental engagement were significant predictors of adolescents meeting recommended MVPA guidelines one year later; however, parental monitoring was not. We also found that the relationships between physical activity and both family cohesion and parent-child communication were mediated by adolescent self-esteem.

Our results are consistent with previous studies demonstrating a direct effect of parenting on children's physical activity. In this study, family cohesion, parental engagement and parent-child communication may reflect one dimension of an authoritative parenting style, which in general includes both responsiveness (provision of emotional support and involvement) and demandingness (provision of appropriate levels of parental control) [23,58]. Schmitz and colleagues found that adolescent girls whose mothers demonstrated an authoritative parenting style reported higher levels of physical activity and lower levels of sedentary behavior [17]. Research on parental influences in tobacco use follows a similar pattern. Parents with authoritative parenting styles, who have household rules against smoking, and who do not smoke are more likely to have adolescents who do not smoke [59]. More cohesive families are also less likely to have adolescents who smoke [60-64]. The lack of association between parental monitoring and physical activity, although inconsistent with studies examining the effects of parental monitoring on other children's health behaviors [65], may reflect other evidence that parenting behaviors which are too directive or restrict children's autonomy are associated with lower levels of child physical activity [25,58]. We also know that parental modeling of physical activity is positively associated with a child's physical activity [6]. Although the Add Health data do not contain information on parental physical activity behaviors, our measure of parental engagement included activities such as playing sports together, providing partial support for the effect of parental physical activity on the child's physical activity. Taken together, these findings suggest the need for additional research on parent influences to design more effective family-focused interventions.

We sought to identify differences in male and female adolescents' reported parenting characteristics and their influence on meeting physical activity guidelines. Overall, there were more similarities than there were differences in regards to predicting MVPA. However, we did find significant gender differences in levels of family cohesion and parent-child communication. Although a higher level of parent-child communication among females is consistent with the literature on adolescent development and family relationships, it is not clear why boys would report higher levels of family cohesion than girls [66]. Despite these differences, we found few gender differences in the influence of parenting characteristics on physical activity. This may be because one of the strongest predictors of physical activity in the logit analysis, parental engagement, did not differ among males and females in the univariate analyses.

Few studies have assessed mediators of parenting influence on adolescent physical activity [67]. Our results provide support for the hypothesis that family cohesion and parent-child communication influence physical activity through their effect on adolescents' emotional health. In both the male and female panels, positive parental relationships were associated with adolescent self-esteem which led to increased physical activity after one year. However, contrary to our hypothesis, we did not observe this same relationship with depression among females. When depressive symptoms were added, family cohesion, parent-child communication and parental engagement remained significant and the strength of the relationship between depressive symptoms and MVPA weakened. The relationship between parental engagement and physical activity, however, was not mediated by self-esteem or depression. Along the continuum of mechanisms of social support and social influences [68], parental engagement may reflect a form of instrumental versus emotional support, the latter of which is likely to have a more direct influence on adolescent's emotional health.

Overall, adolescents in this sample reported having relatively cohesive family relations and moderate levels of parent-child communication, yet very low levels of parental engagement and parental monitoring. This suggests that the adolescents are being raised in a warm household environment with a great deal of autonomy with regards to household rules and guidelines. Therefore, when adolescents are given appropriate levels of autonomy for their developmental stage, they may be better at regulating their own physical activity, as has been suggested by other studies on childhood eating behaviors [69].

Our conclusions must be considered within the context of study limitations. Measures of physical activity, emotional health, and parenting behaviors were all based on adolescent self reports. Given the time period during which these data were collected, an older recommendation for physical activity was used. The use of the 1995 recommendation is likely to overestimate the number of adolescents meeting current physical activity recommendations. The measures of family cohesion and parent-child communication had only moderate levels of internal consistency. The independent and mediating variables were both measured at Wave I, therefore we were not able to establish the temporal relationship between parenting and adolescent emotional health. In addition, one year may be too short a time period to see a causal effect of parenting behaviors on adolescent physical activity. Because physical activity at Wave I was not included as a covariate, we are unable to assess whether previous physical activity levels confounded our results. Despite these limitations, parenting behaviors are likely to be stable over time, suggesting that behaviors reported at Wave I were probably similar to those occurring prior to data collection. Recent studies have shown that peers and the built environment also influence adolescent physical activity [6,70,71]. Our study was not able to control for these factors in our analyses. Further studies should assess the comparative influence of parent support within the context of peer and environmental influences.

## Conclusion

Our findings suggest that a parenting style characterized by warmth and support, while providing adolescents with appropriate levels of autonomy, may be important for achieving recommended levels of physical activity. Together with the results of other studies, our results indicate that an essential component of a health promoting household environment is a well-functioning family system [61-64]. Thus, efforts to engage families to spend time together, communicate with each other, and develop strong family bonds are likely to promote self-esteem and, thereby, physical activity among adolescents.

## Competing interests

The author(s) declare that they have no competing interests.

## Authors' contributions

IO conceived of study, performed the statistical analysis and drafted the manuscript. KP and GA participated in its design and coordination and helped to draft the manuscript. All authors read and approved the final manuscript.

## Supplementary Material

Additional File 1 Correlation matrix for independent variables. The data provided represent a correlation matrix of all independent variables stratified by gender. Click here for file

Additional File 2 Results for Tables 3 and 4 including BMI as a covariate. The data provided represent the analysis presented in Tables 3 and 4, including body mass index as a covariate. Click here for file
