# Supplementary material for: Parental influences on adolescent physical activity: a longitudinal study
Source: Int J Behav Nutr Phys Act. 2007 Feb 2;4:3. doi: 10.1186/1479-5868-4-3 (PMC1805507; doi:10.1186/1479-5868-4-3)
Supplement: Additional File 1 — Correlation matrix for independent variables. The data provided represent a correlation matrix of all independent variables stratified by gender. [file 1479-5868-4-3-S1.doc]

Additional file 1
