# Supplementary material for: Parental influences on adolescent physical activity: a longitudinal study
Source: Int J Behav Nutr Phys Act. 2007 Feb 2;4:3. doi: 10.1186/1479-5868-4-3 (PMC1805507; doi:10.1186/1479-5868-4-3)
Supplement: Additional File 2 — Results for Tables 3 and 4 including BMI as a covariate. The data provided represent the analysis presented in Tables 3 and 4, including body mass index as a covariate. [file 1479-5868-4-3-S2.doc]

Additional file 2
